# Multimodal AI Screening of Developmental Language Disorder in Tunisian Arabic Children: Clinical Markers and Computational Detection

**DOI:** 10.3390/bs16030375

**Published:** 2026-03-06

**Authors:** Faten Bouhajeb, Redha Touati, Selçuk Güven

**Affiliations:** 1 École d’Orthophonie et d’Audiologie, Université de Montréal, Montréal, QC H3T 1J4, Canada; faten.bouhajeb@umontreal.ca; 2 CHU Sainte-Justine Azrieli Research Center, Montréal, QC H3T 1C5, Canada; redha.touati.hsj@ssss.gouv.qc.ca

**Keywords:** developmental language disorder, Tunisian Arabic, clinical assessment, artificial intelligence, machine learning, deep learning, multimodal analysis

## Abstract

Developmental Language Disorder (DLD) is a common neurodevelopmental condition that affects language acquisition in children. However, standardized diagnostic tools for Tunisian Arabic, a widely spoken yet underrepresented dialect, is still lacking. This study presents a multimodal biomedical informatics framework that integrates clinical assessments, speech recordings, and artificial intelligence (AI) for early DLD detection. Three linguistic tasks (the CLT Task, the Arabic Verb Evaluation Task, and the Nonword Repetition Task) were adapted for Tunisian Arabic, and spontaneous speech samples were collected from children with typical development and those with DLD. Statistical analyses revealed significant deficits in verb production, past-tense morphology, and phonological memory in the DLD group. For automated screening, we developed two systems: a Random Forest classifier based on structured clinical and linguistic features and a multimodal deep learning model using Wav2Vec2 acoustic embeddings. The best model achieved an F1 score of 0.85, demonstrating the feasibility of AI-assisted DLD screening. This work introduces the first standardized dataset and computational baseline for DLD in Tunisian Arabic, providing clinically relevant tools for early identification and supporting research on underrepresented Arabic dialects. This work also highlights future implications, including potential applications in early screening, the integration of acoustic markers, and the development of culturally adapted assessment tools for underrepresented languages.

## 1. Introduction

Developmental language disorder (DLD) is characterized by persistent difficulties in acquiring and using language, affecting lexical, grammatical, and phonological domains despite adequate nonverbal intelligence and typical language exposure (Bishop et al., 2017; Leonard, 2014; McGregor, 2020). Early identification is essential for timely intervention and improved long-term educational and social outcomes (Bishop et al., 2017). However, standardized diagnostic tools for underrepresented languages, such as Tunisian Arabic, are lacking, limiting both clinical practice and research.

Arabic dialects, including Tunisian Arabic, exhibit rich morphology, complex verb conjugation, and diglossia, a sociolinguistic situation in which two varieties of the same language coexist with distinct functional roles (e.g., Modern Standard Arabic used in formal contexts and Tunisian Arabic used in daily communication) (Abdalla & Mahfoudhi, 2024). These features complicate direct adaptation of existing DLD assessments, necessitating culturally and linguistically appropriate tools.

Recent advances in artificial intelligence (AI) offer promising approaches for language disorder screening. Machine learning (ML) models can detect patterns in structured clinical data, while deep learning (DL) enables multimodal analysis of audio and textual features, capturing subtle speech and language anomalies often missed in traditional assessments (Dcouto & Pradeepkandhasamy, 2023; Laguarta & Subirana, 2021). By integrating AI with standardized linguistic assessments, our study addresses these limitations, providing a framework that combines structured clinical measures with acoustic analysis to enhance early DLD detection.

This study aims to (i) adapt and standardize clinical linguistic tasks for Tunisian Arabic, (ii) identify discriminative linguistic markers of DLD through clinical and audio analysis, and (iii) develop multimodal AI models to classify children with DLD using combined clinical and acoustic data. To our knowledge, this is the first integration of AI and clinical evaluation for DLD screening in Tunisian Arabic.

### 1.1. Clinical Assessment of DLD

Clinical assessments evaluate lexicon, grammar, and phonology. Lexical tasks, such as the Cross-Linguistic Lexical Task (CLT), assess comprehension and production. The CLT is a standardized tool designed to evaluate receptive and expressive vocabulary (nouns and verbs) across languages, using picture-pointing for comprehension and picture-naming for production. Studies show that children with DLD often struggle more with verbs than nouns (Bedore & Peña, 2008; Khoury Aouad Saliby et al., 2017; Sheng et al., 2012; Simonsen & Haman, 2017). Morphological assessments, exemplified by the Arabic Verb Evaluation Task (AVET), measure verb tense, gender, and number agreement, with prior studies reporting significant challenges in verb morphology for children with DLD (Abdalla & Crago, 2008; Paradis, 2005; Rice & Wexler, 1996; Taha et al., 2021a). Phonological assessments, including Nonword Repetition Tasks (NWRT), reliably indicate short-term phonological memory deficits, a hallmark of DLD across languages (Archibald & Gathercole, 2006; Chiat & Poliszenska, 2016b; Chiat & Polišenská, 2016a).

### 1.2. DLD in Arabic Dialects

Research on Arabic-speaking populations highlights dialectal variability in DLD manifestations. Studies in Palestinian, Hijazi, and Lebanese Arabic indicate that verb morphology, particularly tense and agreement, is a salient marker (Fahim, 2017; Taha et al., 2021b; Tallas-Mahajna et al., 2025). Nonword repetition has also been successfully applied in Arabic, with difficulties amplified by complex syllable structures and consonant clusters (Shaalan, 2020; Taha et al., 2021c). Tunisian Arabic, however, remains underrepresented, and standardized tasks for this dialect have not been systematically validated.

### 1.3. State of the Art and Motivation

AI-driven approaches are increasingly used to assess speech and language disorders, including DLD. Early ML studies analyzed children’s vocal signals to extract phonetic and acoustic descriptors, such as pitch, temporal pauses, articulation dynamics, and prosody, which capture clinically relevant markers (Gong et al., 2016). Advances in automatic speech recognition (ASR) now enable detection of pronunciation errors, articulatory deviations, and atypical speech patterns in neurodevelopmental conditions. However, most ASR systems are optimized for high-resource languages, limiting applicability to underrepresented dialects such as Tunisian Arabic. In the context of AI and language assessment, high-resource languages are those with large annotated corpora, standardized clinical tools, and extensive computational resources (e.g., English, Spanish, Mandarin). Conversely, “low-resource conditions” refer to languages or settings with limited annotated datasets, scarce computational resources, and a lack of standardized assessment tools, which restrict reproducibility and model development. Tunisian Arabic exemplifies such low-resource conditions, highlighting the need for culturally and linguistically adapted diagnostic frameworks.

Acoustic and linguistic analyses have also aided early detection of autism spectrum disorder (ASD) (Li, 2024) and supported clinical decision-making by identifying objective markers of atypical language (Albudoor & Peña, 2022; Quam et al., 2020). Prosodic variation, temporal duration, pitch dynamics, and disfluency patterns complement traditional assessments (Bishop, 2009; Tomas & Vissers, 2018). In adults, AI applications for aphasia severity prediction (Kristinsson et al., 2021), primary progressive aphasia diagnosis (Matias-Guiú et al., 2019), and personalized speech synthesis for dysarthria (Mulfari et al., 2021) are more developed, but developmental disorders in children remain underexplored.

A major limitation is linguistic and data bias: most AI and ASR models are trained on English, with fewer efforts for other high-resource languages such as Spanish or Chinese (Albudoor & Peña, 2022; Lee et al., 2016). Arabic dialects, particularly Tunisian Arabic, remain underrepresented, and the lack of standardized, annotated datasets hampers reproducibility and the development of reliable computational models.

#### 1.3.1. Research Gap

Despite methodological progress, current approaches exhibit three critical limitations (Bouhajeb, 2025): (1) poor generalization to underrepresented or dialectal languages; (2) absence of standardized speech and language datasets for Tunisian Arabic, restricting benchmarking and algorithmic development; and (3) reliance on single-modality pipelines using either clinical measures or acoustic features in isolation, limiting sensitivity to subtle developmental deficits.

#### 1.3.2. Contributions

To address these gaps, we present the first multimodal biomedical informatics framework for automated DLD screening in Tunisian Arabic-speaking children. While traditional clinical assessments provide standardized scores, they may fail to capture subtle acoustic, prosodic, and temporal speech markers present in spontaneous language. Our augmented AI approach addresses these limitations by (i) objectively quantifying acoustic patterns, (ii) integrating linguistic and acoustic cues, (iii) enabling scalable screening in low-resource settings, and (iv) establishing reproducible computational benchmarks. This combination enhances sensitivity and supports more comprehensive early detection of DLD beyond conventional clinical measures. The pipeline integrates standardized linguistic assessments (CLT, AVET, NWRT), fine-grained manual speech annotation, and acoustic representation learning using pretrained Wav2Vec2 embeddings, enabling joint modeling of complementary clinical, linguistic, and prosodic cues.

The study makes three key methodological contributions. First, we provide a culturally adapted assessment pipeline that combines structured clinical–linguistic features with acoustic embeddings to produce interpretable and clinically meaningful diagnostic markers. Second, we systematically compare classical ML models and multimodal deep learning architectures under participant-stratified cross-validation, establishing the first computational benchmark for DLD detection in Tunisian Arabic. Third, leveraging both structured and learned representations yields robust performance in low-resource conditions, offering a transferable blueprint for AI-driven language assessment in other understudied languages. Tunisia presents a unique sociolinguistic landscape, characterized by widespread bilingualism in Arabic and French, and diglossia within Arabic, where Modern Standard Arabic is used in formal contexts and Tunisian Arabic in everyday communication. These cultural and linguistic factors influence language acquisition and assessment, highlighting the need for culturally and linguistically adapted tools when evaluating children for DLD. Together, these contributions advance prior work by addressing linguistic, computational, and clinical challenges in low-resource pediatric populations and providing scalable tools for early DLD screening. In underrepresented languages such as Tunisian Arabic, DLD may be underdiagnosed due to the scarcity of validated assessment tools, limited normative data, and insufficient language-specific markers. These gaps highlight the broader clinical and research relevance of developing culturally and linguistically appropriate diagnostic frameworks.

To guide the study, we explicitly state our research questions. (1) Which lexical, grammatical, and phonological markers most reliably distinguish children with DLD from typically developing peers in Tunisian Arabic? (2) Can multimodal AI models that integrate structured clinical and acoustic features accurately classify DLD in a low-resource language? (3) Does combining structured linguistic features with learned acoustic embeddings improve detection performance?

## 2. Methods

This study combines speech-language evaluation, statistical analysis, and AI-based classification to identify discriminative markers of DLD in Tunisian Arabic-speaking children. Morphological errors reflect deficits in tense and agreement processing, lexical substitutions indicate difficulties in word retrieval and semantic access, and phonological errors, including nonword repetition deficits, point to limitations in phonological working memory. These interpretations are explicitly linked to established findings in DLD literature. All statistical analyses were conducted to examine group differences, interactions, and associations across linguistic domains. Independent-samples t-tests were used to compare children with DLD and typically developing peers, while mixed-design ANOVAs assessed potential interaction effects. Pearson correlations were computed to explore relationships between lexical, grammatical, and phonological measures. Effect sizes were also reported, using Cohen’s d for t-tests and partial $\eta^{2}$ for ANOVAs, to provide a standardized estimate of the magnitude of observed effects. The study administered a range of linguistic tasks to assess multiple domains of language development. Expressive vocabulary was evaluated through picture-naming tasks for nouns and action verbs, while morphosyntactic skills were probed using past-tense sentence completion tasks. Phonological memory was assessed with nonword repetition tasks varying in syllable length and phonotactic complexity. In addition, spontaneous speech samples were elicited using semi-structured narrative prompts to capture naturalistic language use. Lexical, morphological, phonological, and audio-annotation tasks were administered to both DLD and typically developing (TD) children to obtain structured speech-language profiles. Statistical analyses quantified group differences and determined the sensitivity of each speech-language marker. These results informed the development of two complementary classification pipelines: (1) a machine learning model using clinically interpretable linguistic features, and (2) a multimodal deep learning model integrating acoustic, lexical, grammatical and phonological information. All models were trained and evaluated using stratified k-fold cross-validation to evaluate diagnostic accuracy. This integrated approach enables both characterization of language disorder and development of computational tools for automatic DLD detection.

### 2.1. Participant Demographics and Characteristics

Participants were recruited from CHU Sainte-Justine, a university-affiliated pediatric hospital and research center in Montréal, Canada. A total of 42 children aged 3 to 6 years participated, all native speakers of Tunisian Arabic. Evaluations were conducted between October 2024 and February 2025. Children presenting signs of autism spectrum disorder were excluded. All children were exposed to both Tunisian Arabic and French; however, 57.1% primarily spoke Tunisian Arabic at home, whereas 42.9% regularly used both languages. They had varying proficiency levels and underwent an oral language assessment covering vocabulary, grammar, and phonology. The sample included 10 children diagnosed with DLD and 32 TD children. All four children whose parents reported suspected language difficulties were later clinically diagnosed with DLD. The remaining six children with DLD were referred through professional screening rather than parental concern. This observation has been briefly discussed to highlight the potential under-recognition of DLD symptoms by caregivers. DLD diagnosis was confirmed by licensed speech-language pathologists using standardized clinical criteria. All participants had normal hearing and no history of neurological or cognitive impairments (Table 1).

Table 2 presents a summary of the children’s characteristics: age, gender, languages spoken at home, and the profile reported by the parents.

### 2.2. Audio Data Collection, Processing, and Annotation

All sessions were recorded in a quiet room using a high-fidelity microphone. The clinical evaluation lasted approximately 1–1.5 h (including breaks), with an additional 10–20 min dedicated to spontaneous speech sampling. Speech samples were elicited through structured play-based interaction, picture description tasks, and open-ended conversational prompts (e.g., narrating daily activities or describing familiar events). These contexts were chosen to encourage naturalistic expressive language production.

During audio processing, sound levels were first adjusted through harmonization and normalization, followed by the removal of background noise and other artifacts. These steps ensured standardized volume and consistent audio quality across all recordings. We then segmented the audio files to retain only the relevant portions of the child’s speech for reliable downstream analysis.

Manual annotation targeted three main categories of linguistic errors. Manual annotation required approximately 60–90 min per child, depending on speech length and error frequency. All annotations were conducted using ELAN (version 7.0) (ELAN, 2025). The first category included lexical errors, such as substitutions, omissions, or the use of unrelated words. The second category addressed morphological errors, involving incorrect applications of tense, gender, or number. Finally, the third category captured phonological errors, including substitutions and the omission of sounds or syllables. All speech annotations were conducted manually using ELAN (version 7.0). Due to the limited availability of trained annotators for Tunisian Arabic, the full dataset was annotated by a single expert annotator with specialized training in Arabic phonology and morphosyntax. To assess the consistency of the annotation procedure, a subset of recordings (15%) was independently re-annotated by a second trained annotator in the team. Inter-annotator agreement was calculated using Cohen’s kappa for categorical error labels, yielding almost perfect agreement (K = 0.87; (Landis and Koch, 1977)). This verification ensured the reliability of the annotation scheme while maintaining feasibility within a low-resource clinical context.

### 2.3. Clinical Tasks

Three tasks were adapted and administered to assess the lexical, morphological, and phonological domains in Tunisian Arabic.

#### 2.3.1. lightingCLT Task

The CLT evaluates children’s lexical abilities in nouns and verbs through picture-pointing for comprehension and picture-naming for production. Performance was scored for accuracy, and errors were categorized by type. Lexical errors included substitutions (semantic or unrelated), omissions, and circumlocutions; morphological errors included incorrect tense, agreement, or inflection; and phonological errors included substitutions, deletions, and syllable reductions. Effect sizes were calculated using partial eta squared ($\eta_{p}^{2}$).

#### 2.3.2. lightingAVET Task

AVET assesses verb conjugation including tense (past, present), gender (masculine/feminine), and number (singular/plural). Children conjugated verbs in sentence contexts. Scores were calculated per tense, and group differences were evaluated.

#### 2.3.3. lightingNWRT Task

NWR assesses phonological memory by asking children to repeat nonwords of increasing syllable length. The NWRT included items with simple (CV: e.g., “ba”) and slightly more complex (CVC: e.g., “bat”) syllable structures, as well as consonant clusters, balanced across syllable lengths.

### 2.4. Statistical Analysis

Statistical analyses compared the performance of children with DLD and TD peers and identified linguistic markers for AI-based classification. Normality was evaluated using the Shapiro–Wilk test, and homogeneity of variance was assessed with Levene’s test. When assumptions were violated, Welch’s correction was applied. Analyses were performed using Python 3.14.3 with the SciPy 1.17.1 and Statsmodels libraries 0.15.0.

#### 2.4.1. Group Comparability

We assessed whether the groups were comparable in terms of age and gender. Age was assessed with independent-samples Student’s t-tests (Field, 2013), and gender distribution with Chi-square tests (Field, 2013).

#### 2.4.2. Group Differences Across Tasks

Performance on lexical, phonological, and grammatical tasks was compared using independent-samples t-tests with Welch’s correction when needed (Field, 2013). Effect sizes (Cohen’s *d*) were calculated (Cohen, 1988). Pearson correlations examined relationships among linguistic domains.

#### 2.4.3. Mixed-Design ANOVAs

Within-subject factors were analyzed using mixed-design ANOVAs (Field, 2013; Tabachnick & Fidell, 2019). These analyses were applied to tasks involving multiple components, such as comprehension versus production, word class (nouns versus verbs), verb tense (present versus past), agreement features (gender and number), and syllable length. The models evaluated main effects and interactions with group (DLD versus TD). Significant interactions were followed by post hoc tests with Bonferroni correction, enabling fine-grained identification of markers, including difficulties in verb production, past-tense conjugation, and repetition of long nonwords.

#### 2.4.4. Feature Integration for AI Models

Features with significant group effects or strong correlations were used as structured variables in the machine learning classifier and as auxiliary inputs in the multimodal deep learning model.

### 2.5. AI-Based Classification

To enable automated detection of DLD, we implemented two complementary computational approaches: classical machine learning (ML) and multimodal deep learning (DL). Both methods leverage structured clinical measures, manually annotated linguistic features, and acoustic information from children’s speech recordings. By combining structured clinical information with acoustic and linguistic features, this dual approach enables both interpretable feature-based classification (ML) and richer multimodal representation learning (DL), providing complementary insights into automated DLD detection.

#### 2.5.1. Machine Learning (ML)

For the ML approach, we evaluated several classical machine learning models, including Support Vector Machines (SVM), k-Nearest Neighbors (k-NN), and logistic regression. Among these, the Random Forest classifier (Breiman, 2001) yielded the best overall performance and was therefore selected for the final analyses. The Random Forest model was trained on structured features derived from clinical assessment scores and manually annotated linguistic data. This approach provides interpretable classification and enables identification of the most discriminative features contributing to DLD detection through feature-importance analysis.

#### 2.5.2. Multimodal Deep Learning (DL)

The DL approach utilized a multimodal architecture integrating acoustic embeddings and linguistic features. Prosodic and temporal features were modeled automatically through acoustic representation learning rather than manual annotation. Acoustic representations were extracted from the audio recordings using pretrained Wav2Vec2 embeddings, while linguistic features were derived from both clinical scores and annotation data. The network architecture consisted of a feature-fusion layer followed by three fully connected layers with batch normalization and dropout for regularization. The model was trained using a weighted binary cross-entropy loss to account for class imbalance.

#### 2.5.3. Training and Evaluation

All models were evaluated using five-fold stratified cross-validation. Cross-validation folds were created at the participant level, ensuring that all recordings from a given child were confined to a single fold to avoid data leakage. Audio data augmentation included variations in speed and pitch, as well as the addition of background noise. Classification performance was measured using the F1-score. By combining clinical, acoustic, and linguistic features, the models enabled interpretable classification and rich multimodal representation learning.

For the Random Forest baseline, we used 100 trees, no maximum depth, and 7 features considered per split. Class weights were set to balanced to address class imbalance. The model was implemented using scikit-learn.

The DL model was trained for 50 epochs using the Adam optimizer (learning rate = 0.001) with a batch size of 32. Early stopping with a patience of 5 epochs was applied on the validation loss. Weighted binary cross-entropy loss was employed to compensate for class imbalance, with class weights computed from the training set distribution. The model was implemented using PyTorch 2.10.0.

## 3. Experiment Results

In this section, we first present the outcomes of clinical evaluations, highlighting performance across lexical, morphological, and phonological domains. We then identify key linguistic markers that distinguish children with DLD from TD peers in Tunisian Arabic. Finally, we analyze manually annotated audio recordings to examine error patterns in spontaneous speech and report the performance of AI-based models for DLD classification.

### 3.1. Clinical Performance

Participants were evaluated using three tasks: the CLT, the AVET, and the NWRT. These tasks assess core language domains: vocabulary, morphosyntactic skills, and phonological memory, respectively.

#### 3.1.1. lightingCLT Task

The CLT assessed children’s receptive and expressive vocabulary through tasks involving the identification and naming of nouns and verbs. Table 3 summarizes descriptive statistics for each sub-task, word class, and skill type, including means, standard deviations, and minimum and maximum scores.

The first category, comprising the four sub-tasks, shows overall scores ranging from 77% to 93%, with the greatest variability observed in verb production (SD = 18.13%). In terms of word classes, children scored slightly higher on nouns (M = 87%) compared to verbs (M = 84%), regardless of the skill assessed. Finally, regarding skills, comprehension (M = 92%, SD = 8.96%) was consistently higher than production (M = 81%, SD = 14.93%). Comprehension scores (M = 92%) were consistently higher than production (M = 81%), indicating that expressive vocabulary is more vulnerable than receptive skills. Verbs were more challenging than nouns, especially in production (SD = 18.1%), suggesting that verb production deficits may serve as a sensitive lexical marker for DLD.

#### 3.1.2. lightingAVET Task

The AVET assessed participants’ ability to conjugate verbs in Tunisian Arabic, considering tense (present, past), subject-verb agreement in number (singular, plural) and gender (masculine, feminine), and third-person usage. Table 4 presents descriptive statistics.

The global score indicates an average performance of 79% with considerable variability (SD = 24%). Children performed better in present-tense conjugation (M = 94%) than past-tense conjugation (M = 70%). Subject-verb agreement was perfect in singular forms (M = 100%) and slightly lower for plural forms (M = 89%), likely due to increased complexity. Gender agreement scores were high for both masculine (M = 98%) and feminine (M = 94%), and third-person usage was also strong (M = 97%). Overall, the main challenges were conjugating past-tense verbs and managing plural agreement, while person and gender agreement were largely satisfactory. Children performed better on present-tense verbs and singular agreement. Past-tense conjugation (M = 70%) and plural agreement (M = 89%) were more error-prone, suggesting these features as reliable grammatical markers of DLD. High performance in gender and third-person agreement indicates these aspects are less affected in Tunisian Arabic-speaking children with DLD.

#### 3.1.3. lightingNWRT Task

The NWRT evaluated phonological abilities by analyzing overall performance and performance by non-word syllabic length. WRT assessed phonological memory by requiring children to repeat non-words of increasing syllable length. Results are summarized in Table 5. Table 5 presents descriptive statistics.

The overall NWRT score indicates good performance with substantial variability (M = 83%, SD = 19%). Performance declined as syllabic length increased, from 98.8% for two-syllable non-words to 57.7% for five-syllable non-words, with higher variability for longer non-words (SD = 12%). This pattern likely reflects the increased phonological memory load associated with longer and more complex stimuli. Performance declined with increasing syllable length, particularly for four- and five-syllable non-words. This pattern indicates that phonological memory deficits are a robust marker of DLD.

### 3.2. Intergroup Comparisons

A total of ten children were diagnosed with DLD, while thirty-two children were classified as TD. To identify potential linguistic markers of DLD in Tunisian Arabic, comparative analyses were conducted between these two groups. Table 6 summarizes these differences between the two groups.

Prior to examining task performance, we assessed whether the groups were comparable in terms of age and gender. An independent-samples Student’s t-test revealed no significant difference in age between the TD group (M=61, SD=9.8) and the DLD group (M=58, SD=9; t=0.82, p=0.43). Additionally, a Chi-square test indicated no significant difference in gender distribution between the groups ($\chi^{2}\left(1\right)=1.15$, p=0.28), confirming demographic equivalence.

Given the comparability of age and gender, we proceeded with independent-samples t-tests (applying Welch’s correction for unequal variances) to evaluate performance differences between the groups. Across all linguistic tasks, children with DLD performed significantly worse than their TD peers, highlighting the presence of measurable deficits in lexical, grammatical, and phonological domains.

Effect sizes were computed to quantify the magnitude of differences between the DLD and TD groups. The results revealed substantial differences across all linguistic domains, with d=−3.0 for lexical scores, d=−2.41 for phonological scores, and d=−3.76 for grammatical scores. These large effect sizes indicate that the TD and DLD groups are clearly distinguishable, with grammatical performance showing the most pronounced divergence.

Pearson correlation analyses were conducted to examine the interrelationships between the different language measures. Lexical and grammatical scores exhibited a strong positive correlation (r=0.88), indicating substantial shared variance between these domains. Phonological performance showed moderate correlations with lexical (r=0.66) and grammatical scores (r=0.69), suggesting partial overlap while also reflecting distinct aspects of language functioning. Importantly, the identification of lexical and grammatical measures as potential markers of DLD is based on between-group differences and effect sizes rather than on correlation strength alone.

### 3.3. ANOVA Analysis

To identify the specific linguistic components most affected in children with DLD, mixed-design ANOVAs were conducted for each task, examining both within-subject factors and group differences. The larger standard deviations observed in the DLD group reflect greater heterogeneity in performance, consistent with the well-documented variability of DLD phenotypes.

**Lexical (CLT):** For the lexical task (CLT), a mixed-design ANOVA was conducted on word class (nouns vs. verbs) and competence (comprehension vs. production) to determine potential diagnostic markers. The results of the mixed-design ANOVA, including the main effects of group, competence, and word class and their interactions, are summarized in Table 7. A significant main effect of word class was observed, with nouns produced more accurately than verbs (F(1, 40) = 22.4, *p* < 0.001).

The mixed-design ANOVA confirmed that children with DLD performed significantly worse than the control group across all subtasks (*F* values ranging from 71.6 to 75.5, $p<10^{-9}$). A significant group × competence interaction was observed, with the DLD group showing particularly low performance in production tasks (F(1,40)=20.9, $p=4.5\times 10^{-5}$, $\eta_{p}^{2}=0.34$). Additionally, the group × word class interaction indicated that verbs were produced less accurately than nouns by children with DLD (F(1,40)=4.34, p=0.044, $\eta_{p}^{2}=0.10$). The significant Group × Word Class interaction indicates that verb production was disproportionately impaired in the DLD group, supporting its potential role as a sensitive lexical marker. These findings highlight that deficits in expressive language, especially verb production, constitute sensitive markers for identifying DLD in Tunisian Arabic (Table 7).

**Grammatical (AVET):** We conducted similar analyses for the grammatical task (AVET) to examine morphosyntactic performance in both TD and DLD children. Specifically, we focused on the effects of verb tense (present vs. past) and subject-verb agreement in gender (masculine vs. feminine) and number (singular vs. plural). A mixed-design ANOVA was applied to assess the main effects of group, tense, and agreement, as well as their interactions, providing a comprehensive evaluation of how these linguistic factors contribute to differences between DLD and TD participants. The results of this analysis are summarized in Table 8, highlighting significant group effects, significant interactions between group and tense, and the relative contributions of agreement factors to performance variability.

The results reveal a clear and significant distinction between TD and DLD children in the grammatical task. Children with DLD exhibited pronounced difficulties in past-tense verb conjugation compared to present-tense forms, as reflected by a significant group × tense interaction (F(1,40)=32.6, $p=1.2\times 10^{-6}$, $\eta_{p}^{2}=0.45$). In contrast, subject-verb agreement in gender (p=0.065) and number (p=0.64) did not differ significantly between the groups, and no significant interactions involving group and agreement factors were observed. These findings indicate that challenges with past-tense conjugation constitute a robust and specific grammatical marker for identifying DLD in Tunisian Arabic-speaking children.

**Phonological (NWRT):** The phonological abilities of TD and DLD children were further evaluated using the NWRT. The results of a mixed-design ANOVA for this task are presented in Table 9. This analysis examined the main effects of group, syllable length, and their interaction on repetition accuracy. A significant main effect of group was observed (F=45.04, $p=4.74\times 10^{-8}$, $\eta_{p}^{2}=0.53$), indicating that children with DLD consistently performed worse than TD peers across all non-word lengths. Syllable length also showed a strong main effect (F=56.14, $p=9.55\times 10^{-23}$, $\eta_{p}^{2}=0.58$), with accuracy declining as non-word length increased from two to five syllables. Critically, the group × length interaction was significant (F=25.28, $p=9.59\times 10^{-13}$, $\eta_{p}^{2}=0.39$), demonstrating that the performance decrement in DLD children was disproportionately larger for longer non-words, particularly those with four or five syllables. These findings indicate that phonological memory limitations, as reflected by difficulty in repeating longer non-words, constitute a robust and reliable marker of DLD in Tunisian Arabic-speaking children.

Overall, the ANOVA analyses across lexical, grammatical, and phonological tasks consistently highlight the linguistic domains most affected in DLD. Specifically, expressive verb production, past-tense conjugation, and the repetition of long non-words emerge as sensitive and robust markers for the identification of DLD in Tunisian Arabic-speaking children.

### 3.4. Audio Annotation Analysis

Manual annotation of audio recordings revealed that lexical errors, particularly substitutions, were the most frequent errors in the DLD group. Phonological errors also occurred but were less frequent. Morphological errors were mainly observed in past-tense verb conjugation (Table 10). Descriptively, children with DLD produced more spontaneous errors than TD peers across lexical (M = 15.3 vs. 6.2), phonological (M = 7.8 vs. 3.5), and morphological domains (M = 4.1 vs. 1.2), indicating a consistent pattern of increased error production in the DLD group.

### 3.5. AI-Based Classification Results

Both the ML and DL approaches were evaluated for their ability to classify children with DLD versus typically developing (TD) peers. Table 11 summarizes the performance metrics.

**Interpretation:** The Random Forest model achieved the highest F1-score (0.85), outperforming the multimodal DL model (F1 = 0.774). This superior performance of ML can be attributed to the relatively small dataset size, which limits the capacity of DL to fully exploit complex multimodal representations without overfitting. In contrast, ML benefits from structured, manually curated features that are directly informative for DLD classification, leading to robust and interpretable performance.

Although the DL model showed lower overall classification accuracy than models based solely on structured linguistic scores, it integrates acoustic embeddings with linguistic and clinical features within a unified framework. This multimodal configuration allows modeling of prosodic and temporal speech characteristics that are not directly captured by standardized measures. However, in the present sample, the inclusion of acoustic features did not produce a clear incremental improvement in classification performance. Their contribution should therefore be considered exploratory and requires validation in larger samples.

These results suggest that while ML may be more effective on limited, structured datasets, DL provides a complementary perspective that can uncover complex patterns in speech and language. A combined strategy leveraging both approaches may thus maximize accuracy while retaining interpretability and clinical relevance.

### 3.6. Summary of Findings

Overall, the study demonstrates that deficits in verb production, difficulties in past-tense conjugation, and poor performance on longer non-words are reliable markers of DLD in Tunisian Arabic. Lexical substitutions and phonological errors in spontaneous speech further reinforce these markers. Integrating clinical, linguistic, and acoustic features through AI-based classification enables accurate detection of DLD, highlighting the potential of multimodal assessment strategies for clinical and research applications.

## 4. Discussion

This study presents a comprehensive investigation of DLD in Tunisian Arabic by integrating clinical evaluation, linguistic analysis, and AI-based screening. Through the adaptation and standardization of linguistic tasks for this underrepresented dialect, we identified reliable markers of DLD and demonstrated the feasibility of automated multimodal assessment. In bilingual children, particularly those exposed to both Tunisian Arabic and French, careful differentiation between language disorder and typical bilingual variation is essential. The identified markers, especially verb morphology and phonological memory, provide linguistically grounded indicators that are less likely to be confounded by second-language exposure alone. Integrating acoustic analysis with structured linguistic assessment may further assist clinicians in distinguishing persistent developmental deficits from cross-linguistic transfer effects or reduced input in one language. Such tools can therefore support more accurate and equitable diagnostic decision making in bilingual contexts.

### 4.1. Clinical and Linguistic Findings

Lexical deficits, particularly in verb production ($\eta_{p}^{2}=0.34$), emerged as strong indicators of DLD, reflecting demands on lexical retrieval, morphological planning, and syntactic formulation. These observations align with prior work in bilingual and monolingual contexts, including English, Hebrew, and Dutch, where children with DLD demonstrate reduced vocabulary diversity and verb use (Dromi et al., 1999; Leonard, 2014; Rice & Wexler, 1996). Morphological accuracy in past-tense conjugation emerged as a highly sensitive grammatical marker ($\eta_{p}^{2}=0.45$), consistent with findings in other morphologically rich languages such as Hebrew and English (Dromi et al., 1999; Rice & Wexler, 1996), while phonological performance showed a comparable or larger overall effect.

Phonological performance in Nonword Repetition declined sharply with increasing syllable length ($\eta_{p}^{2}=0.55$), indicating reduced phonological memory capacity. This finding is consistent with extensive nonword repetition literature showing that phonological memory deficits are a reliable marker of DLD across languages, including English, Hebrew, and Arabic lighting(Armon-Lotem et al., 2016; Bishop et al., 2017; Gathercole, 2006).

Manual annotation of spontaneous speech further showed that lexical substitutions were the most frequent error type, followed by phonological and morphological deviations. Similar patterns have been observed in studies of bilingual and multilingual children with DLD, suggesting that these error profiles are robust across linguistic contexts (Leonard, 2014; Paradis, 2011). These patterns likely reflect the broader linguistic environment of Tunisian children, characterized by diglossia and multilingual exposure (Ben Youssef & Gries, 2023; Daoud, 2011), without implying direct causal effects on lexical variability.

### 4.2. AI-Based Screening

Machine learning models, including Random Forest, achieved strong performance (F1 = 0.85) using structured clinical and annotated linguistic features. The multimodal deep learning model, combining Wav2Vec2 acoustic embeddings with linguistic features, reached an F1 score of 0.774 and leveraged acoustic representations capable of encoding prosodic and temporal cues such as disfluencies and pauses. These results are in line with recent machine learning studies applying automated speech analysis for DLD detection in languages including Greek, English, and Arabic, highlighting the cross-linguistic potential of AI-based screening tools (Georgiou et al., 2023; Lancaster & Camarata, 2019; Sansavini et al., 2021). Although DLD performance was slightly lower, this approach highlights the methodological potential of acoustic embeddings for detecting subtle language impairments. Together, these findings illustrate how linguistic and acoustic representations provide complementary perspectives for early and culturally appropriate DLD detection.

### 4.3. Clinical and Research Implications

The results confirm that verb production, past-tense morphology, and phonological memory are reliable early markers of DLD in Tunisian Arabic. These markers can support clinicians in developing culturally and linguistically appropriate assessments. AI-based screening tools complement standardized assessments by providing a probabilistic, reproducible classification based on multidimensional features, including acoustic cues not captured by traditional scoring. This approach is scalable to low-resource or large-scale screening contexts. AI is not intended to replace clinical diagnosis but to support early detection and decision-making where full assessment may not be immediately available.

### 4.4. Limitations and Future Directions

This study is limited by a small sample size (DLD = 10, TD = 32) and the use of a single annotator for audio analysis. Future work should increase the sample size across regions, include multiple annotators, and investigate additional linguistic domains such as pragmatics. Further, evaluating cross-dialect generalization will be critical for broad applicability. All children had exposure to both Tunisian Arabic and French, though they differed in primary home language use. This may limit generalization to strictly monolingual Tunisian Arabic speakers. The future work should stratify or control more explicitly for degree of bilingual exposure. Larger datasets will also improve the performance of deep learning models, enabling robust multimodal screening tools for children with DLD. Further research should include conducting longitudinal studies to examine the stability of identified markers over time, exploring additional acoustic and prosodic features to refine detection models, validating the framework using larger multi-site samples, and developing normative databases for Tunisian Arabic to support clinical standardization.

## 5. Conclusions

This study introduced a multimodal framework for screening DLD in Tunisian Arabic by combining standardized clinical assessments with AI-based analysis. Key linguistic markers, including verb production deficits, past tense errors, and reduced phonological memory, were consistently identified, and manual annotations confirmed additional lexical and phonological impairments. Lexical substitutions were significantly more frequent in the DLD group compared to TD peers, reinforcing the role of lexical vulnerability as a distinguishing feature of DLD. Both the Random Forest classifier and the multimodal deep learning model demonstrated strong performance, supporting the feasibility of automated and culturally adapted DLD screening. This work provides clinically relevant assessment tools and establishes the first computational baseline for DLD in Tunisian Arabic. Future work should expand the dataset, refine model architectures, and explore cross-dialect generalization to improve early and accurate detection of DLD in underrepresented languages.

## Figures and Tables

**Table 1:** Participant Demographics.

Group	*n*	Age (Mean ± SD)	Gender (M/F)
DLD	10	5.8 ± 1.0	6/4
TD	32	5.9 ± 0.9	18/14

**Table 2:** Participant Characteristics (*n* = 42).

Variable	*n*	Percentage
Girls	18	42.7%
Boys	24	57.3%
Age 3 to 4 years	2	4.8%
Age 4 to 5 years	13	31.0%
Age 5 to 6 years	19	45.2%
Age 6 to 7 years	8	19.0%
Tunisian Arabic only	24	57.1%
Tunisian Arabic and French	18	42.9%
Typical language development	38	90.5%
Suspected language disorder	4	9.5%

**Table 3:** Lexical performance of participants in the CLT task, reporting mean, standard deviation (SD), minimum, and maximum percentages across comprehension and production of nouns and verbs, as well as overall lexical scores.

Categories	DLD Mean (%)	DLD SD (%)	TD Mean (%)	TD SD (%)	Min (%)	Max (%)
Understanding of Nouns	86	8.9	96	4.2	56	100
Understanding of Verbs	82	9.8	95	4.6	53	100
Production of Nouns	69	17.2	92	6.8	31	100
Production of Verbs	60	18.5	90	7.4	22	94
Nouns	78	12.3	94	5.1	55	100
Verbs	71	13.6	92	5.9	48	97
Understanding	84	9.4	95	4.0	55	100
Production	64	15.1	91	6.3	39	96
Total Lexical Score	68.6	14.7	91.2	4.5	53	98

**Table 4:** Grammatical performance of participants in the AVET task, reporting mean and standard deviation (SD) percentages for global performance and specific grammatical categories.

Score Type	DLD Mean (%)	DLD SD (%)	TD Mean (%)	TD SD (%)	Min (%)	Max (%)
Global Score	43.0	19.5	90.6	8.3	0	100
Verb Tense Score	48	18.7	96	5.2	89.1	94.0
Past	35	22.6	89	9.1	89.1	94.0
Singular	98	4.1	100	0	89.1	94.0
Plural	75	17.8	96	5.4	89.1	94.0
Masculine	90	10.2	99	3.2	89.1	94.0
Feminine	85	11.4	97	4.8	89.1	94.0
Third Person Agreement	92	9.6	99	2.7	89.1	94.0

**Table 5:** Phonological performance of participants in the Nonword Repetition Task (NWRT), reporting mean, standard deviation (SD), minimum, and maximum percentages (%) for each syllable length condition.

Syllable Length	DLD Mean (%)	DLD SD (%)	TD Mean (%)	TD SD (%)	Min (%)	Max (%)
2 syllables	82	14.5	97	3.1	40	100
3 syllables	68	17.2	94	4.8	30	100
4 syllables	54	18.9	90	6.2	20	100
5 syllables	41	19.6	87	7.5	10	100
Total Phonological Score	58.8	16.8	90.0	5.2	25	98

**Table 6:** Comparison of performance scores between children with DLD and TD peers. Mean scores, t-values, and *p*-values are reported. Significance levels are indicated as p<0.001.

Score	DLD Mean	TD Mean	t	*p*
Lexical Score	68.6%	91.2%	−5.89	$1.33\times 10^{-4}$
Phonological Score	58.8%	90%	−4.83	$5.70\times 10^{-4}$
Grammatical Score	43%	90.6%	−6.95	$4.04\times 10^{-5}$

**Table 7:** Analysis of Variance (ANOVA) results for lexical performance, reporting F-values, *p*-values, and partial eta-squared ($\eta_{p}^{2}$). Significance levels are indicated as p<0.001. Separate F-values are reported for noun and verb models where group effects differed slightly across lexical classes.

Effect	F	*p*	$\eta_{p}^{2}$
Group (Nouns)	71.6	<10^−9^	0.64
Group (Verbs)	75.5	<10^−9^	0.64
Competence: Comprehension vs. Production	81.8	3.2 × 10^−11^	0.67
Word Class: Nouns vs. Verbs	22.4	2.7 × 10^−5^	0.36
Group × Competence	20.9	4.5 × 10^−5^	0.34
Group × Word Class	4.34	0.044	0.10

**Table 8:** Analysis of Variance (ANOVA) results for grammatical performance, reporting F-values, *p*-values, and partial eta-squared ($\eta_{p}^{2}$). Significance levels are indicated as p<0.001. Separate F-values are reported for grammatical domains where group effects varied across tense and agreement features.

Effect	F	*p*	$\eta_{p}^{2}$
Group (Tense)	107.1	<10^−4^	0.73
Group (Agreement: Gender)	24.1	<10^−4^	0.33
Tense: Present vs. Past	39.1	2.1 × 10^−7^	0.49
Number Agreement: Singular vs. Plural	0.23	0.64	0.006
Gender Agreement: Masculine vs. Feminine	3.6	0.065	0.08
Group × Tense	32.6	1.2 × 10^−6^	0.45
Group × Gender	1.8	0.18	<0.05
Group × Number	1.8	0.18	<0.05

**Table 9:** Analysis of Variance (ANOVA) results for phonological performance, reporting F-values, *p*-values, and partial eta-squared ($\eta_{p}^{2}$). Significance level: p<0.001.

Effect	F	*p*	$\eta_{p}^{2}$
Group: DLD vs. TD	45.04	$4.74\times 10^{-8}$	0.53
Length (2 to 5 syllables)	56.14	$9.55\times 10^{-23}$	0.58
Group × Length Interaction	25.28	$9.59\times 10^{-13}$	0.39

**Table 10:** Mean ± standard deviation (SD) of annotated speech errors per domain for children with DLD and typically developing (TD) peers.

Error Type	DLD Mean ± SD	TD Mean ± SD
Lexical Substitutions	15.3 ± 3.2	6.2 ± 1.7
Phonological Errors	7.8 ± 2.0	3.5 ± 1.2
Morphological Errors	4.1 ± 1.4	1.2 ± 0.6

**Table 11:** F1-score performance of AI models for DLD classification, evaluated using five-fold stratified cross-validation with audio data augmentation.

Model	F1-Score
Random Forest (ML)	0.85
Multimodal Deep Learning	0.774

## Data Availability

The data presented in this study are not publicly available due to ethical and privacy restriction.

## References

[B1-behavsci-16-00375] Abdalla F., Crago M. (2008). The acquisition of verb morphology in Arabic-speaking children with specific language impairment. Clinical Linguistics & Phonetics.

[B2-behavsci-16-00375] Abdalla F., Mahfoudhi A. (2024). Verb agreement production in Arabic-speaking children with developmental language disorder. Language Acquisition.

[B3-behavsci-16-00375] Albudoor N., Peña E. D. (2022). Identifying language disorder in bilingual children using automatic speech recognition. Journal of Speech, Language, and Hearing Research.

[B4-behavsci-16-00375] Archibald L. M. D., Gathercole S. E. (2006). Nonword Repetition: A Comparison of Tests. Journal of Speech, Language, and Hearing Research.

[B5-behavsci-16-00375] Armon-Lotem S., Edwards J., Meir N. (2016). Assessing multilingual children: Disentangling bilingualism from language impairment.

[B6-behavsci-16-00375] Bedore L. M., Peña E. D. (2008). Assessment of bilingual children for identification of language impairment: Current findings and implications for practice. International Journal of Bilingual Education and Bilingualism.

[B7-behavsci-16-00375] Ben Youssef C., Gries S. T. (2023). Code-switching in Tunisian Arabic: A multi-factorial random forest analysis. Corpora.

[B8-behavsci-16-00375] Bishop D. V. M. (2009). Children who read words accurately despite language impairment: Who are they and how do they do it?. Child Development.

[B9-behavsci-16-00375] Bishop D. V. M., Snowling M. J., Thompson P. A., Greenhalgh T., CATALISE-2 Consortium, House A. (2017). Phase 2 of CATALISE: A multinational and multidisciplinary Delphi consensus study of problems with language development: Terminology. Journal of Child Psychology and Psychiatry.

[B10-behavsci-16-00375] Bouhajeb F. (2025). L’intelligence artificielle: Un moyen de dépistage des troubles du langage chez les enfants arabophones. Master’s thesis.

[B11-behavsci-16-00375] Breiman L. (2001). Random forests. Machine Learning.

[B12-behavsci-16-00375] Chiat S., Poliszenska J. (2016). Assessing language impairment in bilingual children: Methodological issues and clinical implications. International Journal of Language & Communication Disorders.

[B13-behavsci-16-00375] Chiat S., Polišenská K. (2016). A framework for crosslinguistic nonword repetition tests: Effects of bilingualism and socioeconomic status on children’s performance. Journal of Speech, Language, and Hearing Research.

[B14-behavsci-16-00375] Cohen J. (1988). Statistical power analysis for the behavioral sciences.

[B15-behavsci-16-00375] Daoud M. (2011). The sociolinguistic situation in Tunisia: Language rivalry or accommodation?. International Journal of the Sociology of Language.

[B16-behavsci-16-00375] Dcouto S. S., Pradeepkandhasamy J. (2023). Multimodal deep learning in early autism detection—Recent advances and challenges. Engineering Proceedings.

[B17-behavsci-16-00375] Dromi E., Leonard L. B., Adam G., Zadunaisky-Ehrlich S. (1999). Verb agreement morphology in Hebrew-speaking children with specific language impairment. Journal of Speech, Language, and Hearing Research.

[B19-behavsci-16-00375] ELAN (2025). Elan *(Version 7.0) [Computer software]*.

[B20-behavsci-16-00375] Fahim D. (2017). Verb morphology in Egyptian Arabic developmental language impairment. Arab Journal of Applied Linguistics.

[B21-behavsci-16-00375] Field A. (2013). Discovering statistics using IBM SPSS Statistics.

[B22-behavsci-16-00375] Gathercole S. E. (2006). Nonword repetition and word learning: The nature of the relationship. Applied Psycholinguistics.

[B18-behavsci-16-00375] Georgiou G. P., Theodorou E. (2024). Detection of developmental language disorder in Cypriot Greek children using a neural network algorithm. Journal of Technology in Behavioral Science.

[B23-behavsci-16-00375] Gong J., Gong M., Levy-Lambert D., Green J., Hogan T., Guttag J. (2016). Towards an automated screening tool for developmental speech and language impairments. Interspeech 2016.

[B24-behavsci-16-00375] Khoury Aouad Saliby C., dos Santos C., Kouba Hreich E., Messarra C. (2017). Assessing Lebanese bilingual children: The use of cross-linguistic lexical tasks in Lebanese Arabic. Clinical Linguistics & Phonetics.

[B25-behavsci-16-00375] Kristinsson S., Zhang W., Rorden C., Newman-Norlund R., Basilakos A., Bonilha L., Yourganov G., Xiao F., Hillis A. E., Fridriksson J. (2021). Machine learning–based multimodal prediction of language outcomes in chronic aphasia. Human Brain Mapping.

[B26-behavsci-16-00375] Laguarta J., Subirana B. (2021). Longitudinal speech biomarkers for automated Alzheimer’s detection. Frontiers in Computer Science.

[B27-behavsci-16-00375] Lancaster H. S., Camarata S. (2019). Reconceptualizing developmental language disorder as a spectrum disorder: Issues and evidence. International Journal of Language & Communication Disorders.

[B28-behavsci-16-00375] Landis J. R., Koch G. G. (1977). The measurement of observer agreement for categorical data. Biometrics.

[B29-behavsci-16-00375] Lee T., Liu Y., Huang P.-W., Chien J.-T., Lam W. K., Yeung Y.-T., Law T. K.-T., Lee K. Y. S., Kong A. P.-H., Law S.-P. (2016). Automatic speech recognition for acoustical analysis and assessment of Cantonese pathological voice and speech. 2016 IEEE international conference on acoustics, speech and signal processing (ICASSP).

[B30-behavsci-16-00375] Leonard L. B. (2014). Children with specific language impairment.

[B31-behavsci-16-00375] Li J. (2024). Artificial intelligence-based detection of autism spectrum disorder using linguistic features. 2024 IEEE 3rd international conference on computing and machine intelligence (ICMI).

[B32-behavsci-16-00375] Matias-Guiú J. A., Díaz-Álvarez J., Cuetos F., Cabrera-Martín M. N., Segovia-Ríos I., Pytel V., Moreno-Ramos T., Carreras J. L., Ayala J. L. (2019). Machine learning in the clinical and language characterisation of primary progressive aphasia variants. Cortex.

[B33-behavsci-16-00375] McGregor K. K. (2020). How we fail children with developmental language disorder. Language, Speech, and Hearing Services in Schools.

[B34-behavsci-16-00375] Mulfari D., Meoni G., Marini M., Fanucci L. (2021). Machine learning assistive application for users with speech disorders. Applied Soft Computing.

[B35-behavsci-16-00375] Paradis J. (2005). Grammatical morphology in children learning English as a second language. Language, Speech, and Hearing Services in Schools.

[B36-behavsci-16-00375] Paradis J. (2011). Individual differences in child English second language acquisition: Comparing child-internal and child-external factors. Linguistic approaches to bilingualism.

[B37-behavsci-16-00375] Quam C., Cardinal H., Gallegos C., Bodner T. (2020). Sound discrimination and explicit mapping of sounds to meanings in preschoolers with and without developmental language disorder. International Journal of Speech-Language Pathology.

[B38-behavsci-16-00375] Rice M. L., Wexler K. (1996). Toward tense as a clinical marker of specific language impairment in English-speaking children. Journal of Speech and Hearing Research.

[B39-behavsci-16-00375] Sansavini A., Favilla M. E., Guasti M. T., Marini A., Millepiedi S., Di Martino M. V., Vecchi S., Battajon N., Bertolo L., Capirci O., Carretti B., Colatei M. P., Frioni C., Marotta L., Massa S., Michelazzo L., Pecini C., Piazzalunga S., Pieretti M., Lorusso M. L. (2021). Developmental language disorder: Early predictors, age for the diagnosis, and diagnostic tools: A scoping review. Brain Sciences.

[B40-behavsci-16-00375] Shaalan S. (2020). Nonword repetition skills in Gulf Arabic–speaking children with developmental language disorder. Journal of Speech, Language, and Hearing Research.

[B41-behavsci-16-00375] Sheng L., Peña E. D., Bedore L. M., Fiestas C. E. (2012). Semantic deficits in Spanish–English bilingual children with language impairment. Journal of Speech, Language, and Hearing Research.

[B42-behavsci-16-00375] Simonsen H., Haman E. (2017). LITMUS-CLT: A new way to assess bilingual lexicons. Clinical Linguistics & Phonetics.

[B43-behavsci-16-00375] Tabachnick B. G., Fidell L. S. (2019). Using multivariate statistics.

[B44-behavsci-16-00375] Taha J., Stojanovik V., Pagnamenta E. (2021a). Sentence repetition as a clinical marker of developmental language disorder: Evidence from Arabic. Journal of Speech, Language, and Hearing Research.

[B45-behavsci-16-00375] Taha J., Stojanovik V., Pagnamenta E. (2021b). Expressive verb morphology deficits in Arabic-speaking children with developmental language disorder. Journal of Speech, Language, and Hearing Research.

[B46-behavsci-16-00375] Taha J., Stojanovik V., Pagnamenta E. (2021c). Nonword repetition performance of Arabic-speaking children with and without developmental language disorder. Journal of Speech, Language, and Hearing Research.

[B47-behavsci-16-00375] Tallas-Mahajna N., Armon-Lotem S., Saiegh-Haddad E. (2025). The emergence of verb patterns in Arabic in children with developmental language disorder compared to children with typical development. Journal of Speech, Language, and Hearing Research.

[B48-behavsci-16-00375] Tomas E., Vissers C. (2018). Behind the scenes of developmental language disorder: Time to call neuropsychology back on stage. Frontiers in Human Neuroscience.

